# Supplementary material for: The Impact of Cannabidiol (CBD) on Lipid Absorption and Lymphatic Transport in Rats
Source: Nutrients. 2025 Mar 15;17(6):1034. doi: 10.3390/nu17061034 (PMC11944757; doi:10.3390/nu17061034)
Supplement: Supplementary file 1 [file nutrients-17-01034-s001.zip › nutrients-3522913-supplementary.pdf]

**Supplemental Figures**.

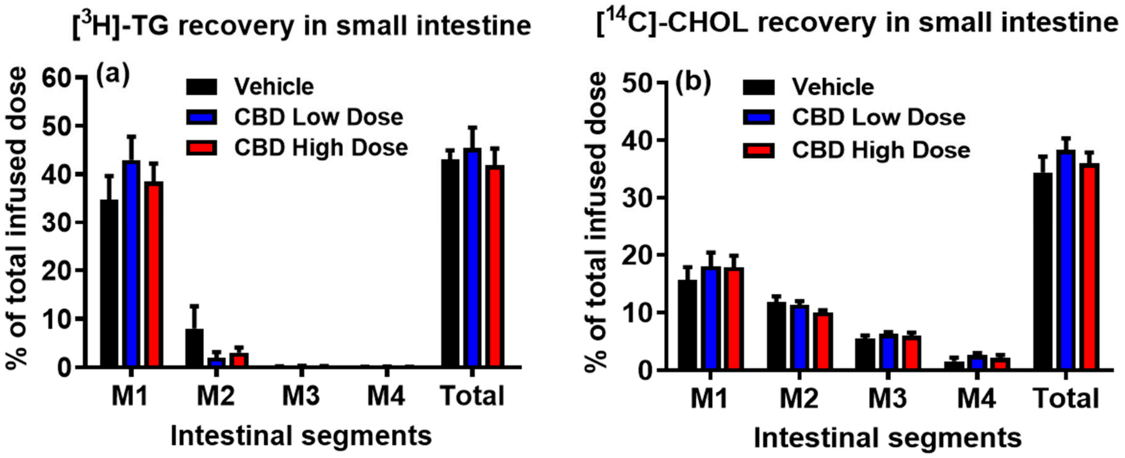


**Figure S1.** Distribution of [$^{3}$H]-TG (a) and [$^{14}$C]-CHOL (b) across small intestine segments. Radioactivity was determined by a scintillation counter. Data are presented as means ± SEM. Vehicle group: black column (n=8); low-dose CBD group (10 mg/kg): blue column (n=10); and high-dose CBD group (30 mg/kg): red column (n=10).

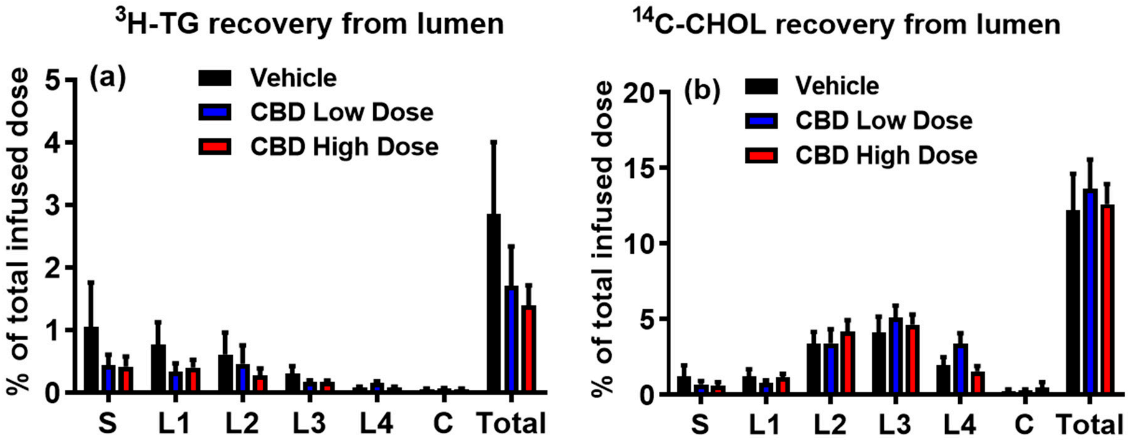


**Figure S2.** Comparison of [$^3$H]-TG (a) and [$^{14}$C]-CHOL (b) remained in the lumen after 6 hours of treatment with or without CBD. Luminal contents were collected from the stomach (S), four equal segments of the small intestine (L1–L4, starting from the duodenum), and the colon (C). L1 corresponds to M1 (duodenum), L2 and L3 to M2 and M3 (jejunum), and L4 to M4 (ileum). No significant differences in [$^3$H]-TG (a) or [$^{14}$C]-CHOL (b) accumulation were observed among the three groups. Data are presented as mean ± SEM. Vehicle group: black columns (n=8); low-dose CBD (10 mg/kg): blue columns (n=10); high-dose CBD (30 mg/kg): red columns (n=10).

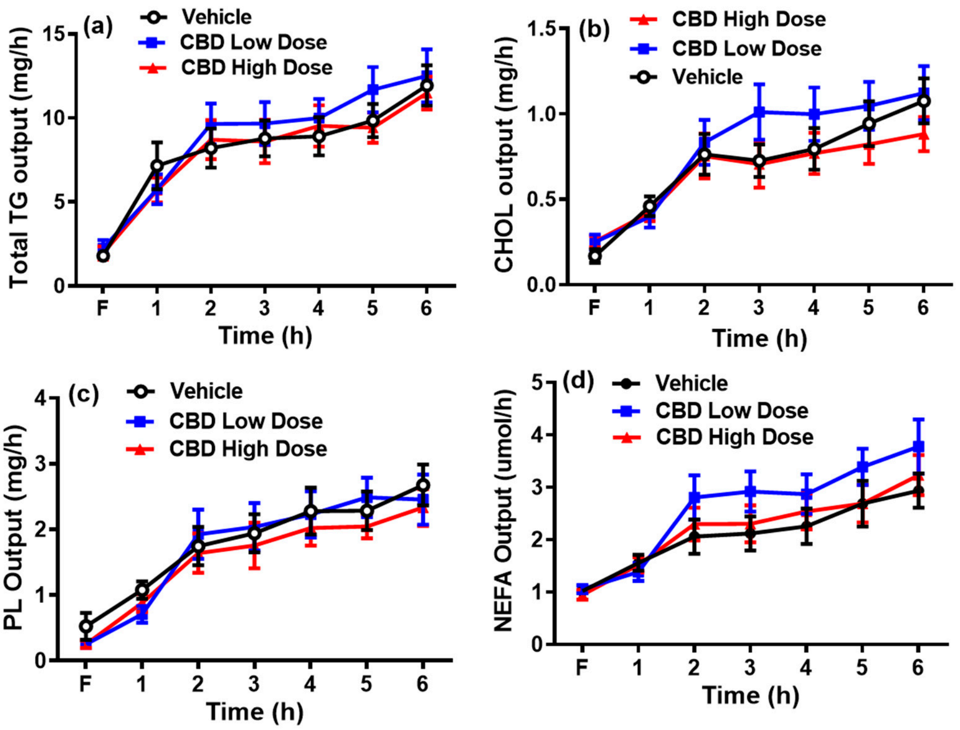


**Figure S3**. Comparison of lymphatic output of total TG mass (a), CHOL mass (b), PL mass (c), and NEFA mass (d). Data are presented as mean ± SEM. Vehicle group: black circles (n=8); low-dose CBD group (10 mg/kg): blue squares (n=10); high-dose CBD group (30 mg/kg): red triangles (n=10).

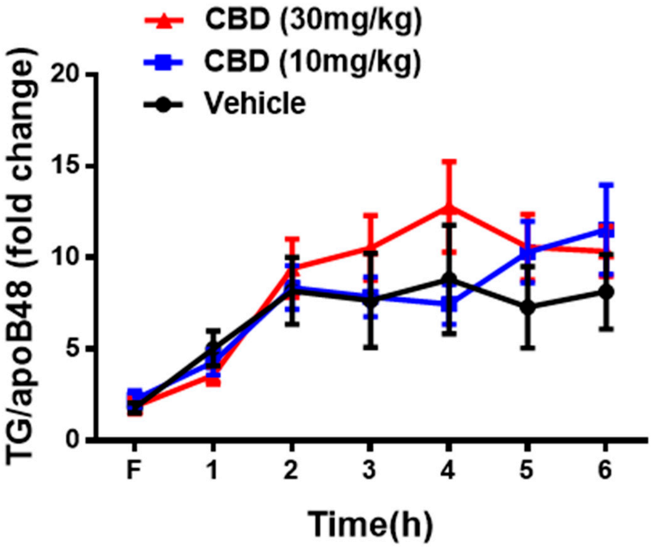


**Figure S4:** Fold-change in the ratio of lymph triglyceride output (TG) to apoB48 output relative to the time of treatment. Values are means ± SEM. Vehicle group: black squares (n=8); low-dose CBD group (10 mg/kg): blue circles (n=10); high-dose CBD group (30 mg/kg): red triangles (n=10).
